# Building Information Modeling (BIM) Driven Carbon Emission Reduction Research: A 14-Year Bibliometric Analysis

**DOI:** 10.3390/ijerph191912820

**Published:** 2022-10-06

**Authors:** Zhen Liu, Peixuan Li, Fenghong Wang, Mohamed Osmani, Peter Demian

**Affiliations:** 1 School of Design, South China University of Technology, Guangzhou 510006, China; 2 School of Architecture, Building and Civil Engineering, Loughborough University, Loughborough LE11 3TU, UK

**Keywords:** building information modeling (BIM), carbon emissions, China, sustainable building, building metaverse, lifecycle, design, policy, visualization, bibliometric

## Abstract

Governments across the world are taking actions to address the high carbon emissions associated with the construction industry, and to achieve the long-term goals of the Paris Agreement towards carbon neutrality. Although the ideal of the carbon-emission reduction in building projects is well acknowledged and generally accepted, it is proving more difficult to implement. The application of building information modeling (BIM) brings about new possibilities for reductions in carbon emissions within the context of sustainable buildings. At present, the studies on BIM associated with carbon emissions have concentrated on the design stage, with the topics focusing on resource efficiency (namely, building energy and carbon-emission calculators). However, the effect of BIM in reducing carbon emissions across the lifecycle phases of buildings is not well researched. Therefore, this paper aims to examine the relationship between BIM, carbon emissions, and sustainable buildings by reviewing and assessing the current state of the research hotspots, trends, and gaps in the field of BIM and carbon emissions, providing a reference for understanding the current body of knowledge, and helping to stimulate future research. This paper adopts the macroquantitative and microqualitative research methods of bibliometric analysis. The results show that, in green-building construction, building lifecycle assessments, sustainable materials, the building energy efficiency and design, and environmental-protection strategies are the five most popular research directions of BIM in the field of carbon emissions in sustainable buildings. Interestingly, China has shown a good practice of using BIM for carbon-emission reduction. Furthermore, the findings suggest that the current research in the field is focused on the design and construction stages, which indicates that the operational and demolition stages have greater potential for future research. The results also indicate the need for policy and technological drivers for the rapid development of BIM-driven carbon-emission reduction.

## 1. Introduction

The issue of global warming caused by carbon dioxide (CO_2_) emissions is a concern for countries across the world [1]. The Paris Agreement, signed at the Paris Climate Conference in year 2015, led to a consensus among several countries to aim to limit the global average warming to 2 °C above the preindustrial levels, and to work towards limiting it to below 1.5 °C [2]. To achieve this goal, the Special Report on Global Warming of 1.5 °C from the Intergovernmental Panel on Climate Change (IPCC) emphasizes that, to limit warming to 1.5 °C, global carbon emissions need to be halved by year 2030, and a carbon-neutrality target needs to be achieved by mid-century [3]. The construction industry accounts for a significant proportion of the world’s energy consumption and carbon emissions, of which the construction and operation of buildings represent 36% of the total final energy use, and nearly 40% of the greenhouse gas (GHG) emissions [4]. Therefore, reducing the energy consumption and carbon emissions of buildings has great significance for environmental protection and sustainable development [5,6]. At present, governments around the world are taking actions to address the problems related to the high carbon emissions of the construction industry, and they are striving to achieve the carbon-neutrality goal [7,8].

The application of building-information-modeling (BIM) technology brings about new possibilities for the development of sustainable buildings, for which predicting, managing, and monitoring the impact of buildings on the environment through digital technology can improve the design process [9]. Meanwhile, the use of BIM has the potential to improve the energy efficiency of sustainable buildings and reduce carbon emissions, which results in the provision of people with healthy and comfortable living environments [10]. In addition, BIM is at the heart of the digital transformation of the construction industry, and it supports digital twins by acting as a data-management platform [11]. Augmented reality (AR) and virtual reality (VR) technologies are now increasingly being used to enhance the adoption of BIM [12], which seems to a trend towards a building metaverse.

Research into minimizing the energy consumption associated with carbon emissions throughout the lifecycles of buildings is rapidly being developed [13]. However, it has been argued that the previous studies related to carbon emissions lacked in-depth investigation [14]. At present, the studies on BIM and carbon emissions have concentrated on the design stage of the building lifecycle, with topics focusing on the use of the building energy and the calculation of the associated carbon emissions [15,16,17,18]. However, the effect of BIM in reducing the carbon emissions over the building lifecycle phases is not well researched. Reducing building carbon emissions is of great significance to achieving the carbon-neutrality goal. As such, an increasing number of researchers are calling for investigations into the impact of the BIM application on reducing carbon emissions across all the building lifecycle stages [19]. Hence, the aim of this paper is to explore the relationship between BIM and carbon emissions within the context of sustainable buildings by reviewing and assessing the current research hotspots and trends in the use of BIM for carbon-emission reductions across all the lifecycle stages of buildings, which provides a useful reference for understanding the current body of knowledge, and stimulates future research.

## 2. Materials and Methods

This study adopted a mixed-research approach that comprised: (1) accessing relevant publications from the Web of Science (WoS) database; (2) exploring the relationship between BIM, carbon emissions, and sustainable buildings from both the macroquantitative and microqualitative perspectives.

In the macroquantitative analysis, bibliometric methods were used to identify the research structures and themes. The research methodology of bibliometric analysis provides an objective and straightforward demonstration of the characteristics and trends of existing research, informing and inspiring more in-depth studies [20]. First, the results-analysis tools in the WoS database were used to analyze the number and sources of BIM publications in the field of sustainable-building carbon emissions in the form of bar and pie charts in order to obtain the current research progress and research trends. Second, the literature was visually analyzed for the keywords, taking advantage of the different features of the bibliometric visualization software from VOSviewer and CiteSpace. VOSviewer is a software tool for building and visualizing bibliometric networks. VOSviewer provides text-mining capabilities that can be used to build and visualize co-occurrence networks of important terms extracted from large volumes of scientific literature. In addition, VOSviewer’s interface is easy to operate and more suitable for identifying and analyzing topic clusters [21]. Because the keywords of the literature can distil the core content of the literature, the keyword-co-occurrence-analysis method can well reflect the current academic research hotspots, knowledge structures, and development trends of some disciplines [22]. The keyword-co-occurrence method has been applied to many research fields, such as medicine, environmental science, and engineering [23,24,25,26]. Due to the large timespan of the literature search, VOSviewer’s presentation of the research hotspots in the temporal dimension is not ideal, but CiteSpace’s burst-detection-analysis function compensates for this shortcoming. Based on CiteSpace’s keyword-burst detection, it is possible to identify the burst keywords for each year of the research period, as well as to better analyze the research hotspots and trends in the temporal dimension [27], thus combining the visual-measurement software of the literature in a macroanalysis.

Due to the complexity of scientific development, the use of bibliometrics can only provide a general analysis of the laws of scientific development. To fill in the gaps in the macroanalysis, and based on the findings of the macroquantitative analysis and the purpose of this research paper, a microqualitative analysis was conducted. In the microanalysis, the publications searched in the WoS database were first reviewed, and the strong relevant literature on BIM in the field of sustainable carbon emissions was identified. This was followed by the mapping of the strongly relevant literature to the building lifecycle, and a comprehensive analysis of the impact of BIM on building carbon emissions at all stages of the building lifecycle, to obtain more knowledgeable contributions.

The quantity and quality of the data have a significant impact on the results of bibliometric visualization. To ensure the comprehensiveness and higher reliability of the research data, one of the largest and most important international databases, WoS, was used as the data source for this study [28]. Additionally, to ensure the quality of the articles, the WoS core collection was selected, for which the indexed results were set as journal articles and reviews. The search was based on topics, with keywords such as “BIM”, “building information model*”, “carbon emissions”, “carbon trading”, “carbon credits”, “low carbon”, “sustainable building”, and “green building”. 

**Figure 1:**
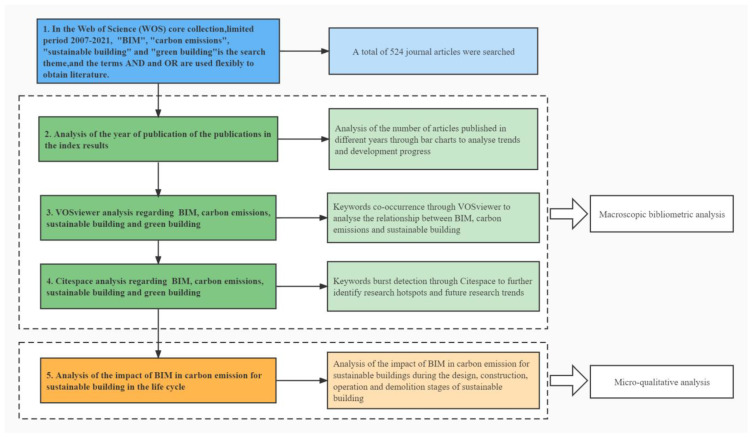
Research method flow diagram.

The research-method flow is illustrated in Figure 1, and it comprises five steps: (1) a search by topic in the WoS core collection to collect data; (2) an analysis of the current status of the publications through bar charts and pie charts; (3) a keyword-co-occurrence analysis through VOSviewer software, including network visualization and high-frequency keywords; (4) keyword-burst detection through CiteSpace software; (5) a lifecycle-based analysis of the impact of BIM on carbon-emission reduction within the context of sustainable buildings.

## 3. Results

### 3.1. Number of Publications and Sources of Publications in the Field of Building Information Modeling (BIM), Carbon Emissions, and Sustainable Buildings

**Figure 2:**
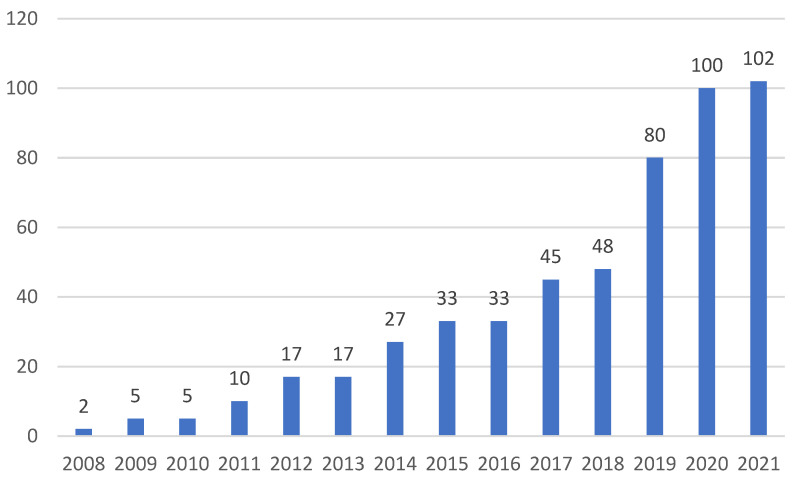
Number of articles published in each year on BIM in the field of carbon emissions within the context of sustainable buildings from 2008 to 2021 (14 years) in the Web of Science (WoS) core collection (generated by the authors).

The keyword searches, such as “BIM”, “building information model*”, “carbon emissions”, “carbon trading”, “carbon credits”, “low carbon”, “sustainable building”, and “green building”, generated a total of 524 results for the period from 2008 to 2021 (14 years), of which the first related study in the WoS database is from year 2008. As shown in Figure 2, the graph of the publication statistics shows that the number of publications on BIM associated with carbon emissions and sustainable buildings slowly increased from year 2008 (2 articles) to 2018 (48 articles). Between the years 2018 and 2020, the number of publications showed a rapid increase, of which the total 100 published articles in the year 2020 was more than twice the number of publications compared with 2018. Interestingly, almost the same number (102) of articles were published in year 2021 and 2020, which indicates that studies that investigate the relationship between BIM and carbon emissions for sustainable buildings have become an emerging scheme. 

**Figure 3:**
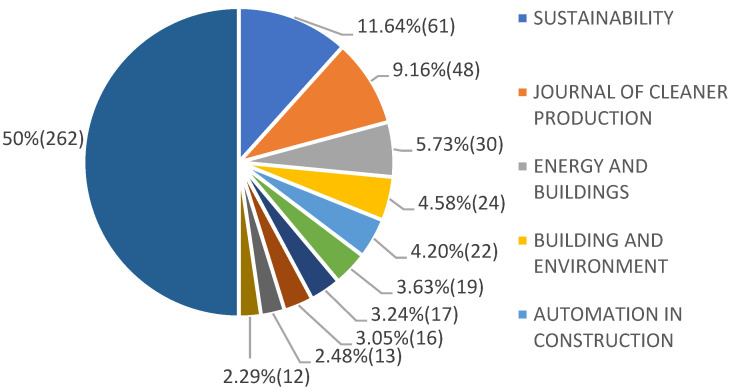
Sources of published articles regarding BIM on carbon emissions for sustainable buildings from 2008 to 2021 (14 years) in WoS (generated by the authors).

The sources of the BIM publications in the area of carbon emissions within the context of sustainable buildings are shown in Figure 3. A total of 524 articles have been published in 150 journals, of which 50% of all the published articles are from 10 leading sources, which are: Sustainability, with 11.64% of all the published articles, followed by Journal of Cleaner Production (9.16%), Energy and Buildings (5.73%), Building and Environment (4.58%), Automation in Construction (4.20%), Sustainable Cities and Society (3.63%), Renewable Sustainable Energy Reviews (3.24%), Energies (3.05%), Building Research and Information (2.48%), and Buildings (2.29%).

### 3.2. Results of Macroquantitative Analysis

The 524 articles were imported into the software VOSviewer (version 1.6.17) to generate a term diagram of the co-clustering for the keyword-co-occurrence analysis. A total of 2434 keywords were generated, from which the minimum threshold number of the keyword occurrence was 7 times, encompassing 112 keywords that met the threshold. They are displayed using network and overlay visualization.

#### 3.2.1. Network Visualization of BIM in the Field of Carbon Emissions for Sustainable Buildings

**Figure 4:**
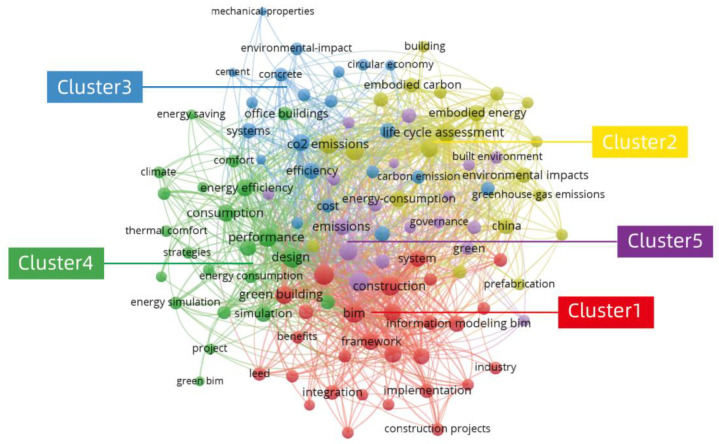
Keyword-network visualization of published articles regarding BIM in the field of carbon emissions for sustainable buildings from 2008 to 2021 (14 years) via VOSviewer software (generated by the authors).

Figure 4 shows a network-visualization map of the keyword co-occurrence in the field of BIM and carbon emissions for sustainable buildings. In network visualization, there are text labels, circles, connections, and color areas. By default, the items are represented by the text labels and circles. The size of the circle indicates the frequency of the occurrence of a keyword. The larger the circle, the more frequently the keyword appears. The distance between the positions of two items and the thickness of the connecting lines represent the strength of their direct affinity [29]. In addition, the more lines there are, the more co-occurrence there is between the keywords. At the same time, the different colored areas represent different clusters, and this view allows each individual cluster to be viewed [30]. Based on the network-visualization map of the keyword co-occurrence, it is possible to analyze the knowledge structure and research hotspots of BIM in the field of carbon emissions in sustainable buildings.

Cluster 1 (red color), with the theme of sustainable-building construction, including “BIM”, “construction”, “sustainability”, “green building”, and “framework”, indicates that close links have been established between BIM and green building or sustainable construction. Cluster 2 (yellow color) is associated with the building lifecycle assessment, including “life cycle assessment”, “carbon emissions”, “energy consumption”, “embodied energy”, and “environmental impacts”. Cluster 3 (blue color) relates to sustainable-building materials, including “buildings”, “systems”, “efficiency”, “concrete”, and “cement”. Cluster 4 (green color) is focused on the sustainable-building energy efficiency, with its “design”, “performance”, “consumption”, and “energy efficiency”. Cluster 5 (purple color) is correlated to environmental strategies, including “energy”, “green buildings”, “policy”, “emissions”, “challenges”, “governance”, “sustainable development”, and “climate change”.

“BIM”, “Green building”, and “sustainability” are very closely linked in Cluster 1 (red color). The distances between the keywords in the network-visualization map roughly indicate their relevance in the co-occurrence network, in which the closer the position of two items, the stronger their correlation in terms of the occurrence links in the group of publications analyzed. The terms “green building” and “sustainable building” are deemed as two terms with the same meaning [31]. The development of green buildings and standards are instrumental in achieving the national targets for reducing carbon emissions, for which BIM provides an important technical support to achieve the green-building concepts [32]. In addition, BIM contributes to the development of environmental-performance-evaluation systems for green buildings, accelerating the process of the building assessment [33,34,35], such as Leadership in Energy and Environmental Design (LEED), which assists designers to evaluate the sustainability solutions during the conceptual design stage [36,37,38,39]. Furthermore, the role of BIM in supporting the carbon-emission calculation for green buildings greatly promotes the implementation and development of energy-conservation and emission-reduction policies [40]. It is worth focusing on the fact that, although the concept of sustainable buildings has been proposed for a long time, it has mainly been promoted and applied in developed countries, while the promotion in developing countries still faces problems associated with technology, funding, and energy [41], which encounter challenges similar to the current implementation of BIM. 

In Cluster 2 (yellow color), there is a strong link between “Life Cycle Assessment (LCA)”, “carbon emissions”, and “carbon footprint”. To enhance and measure the sustainability performance in the construction industry, the LCA can be used to assess the environmental impacts of buildings throughout all the lifecycle stages [42]. The integrated tool based on BIM and LCA can calculate carbon emissions during the lifecycle of a building, and it can thus help to set carbon-emission targets and sustainable policies [18,43,44,45]. In addition, the BIM-enhanced LCA system can be used to quickly assess building design solutions and enable sustainable decision making [43,46,47]. Furthermore, LCA-based BIM can support the selection of sustainable products and materials during the building design and construction stages, thereby improving the sustainability of the building [48].

In Cluster 3 (blue color), “concrete”, “cement,” and “buildings” are very closely associated with each other. The choice of building material has a significant impact on the building performance [49]. Concrete and its main material, cement, are the main building materials in the world, but the production of cement produces large amounts of CO_2_, which is not conducive to sustainable development in the construction industry [50,51,52]. With the development of new technologies and the invention of new materials, there are opportunities to reduce the carbon emissions from cement [53,54]. Prefabricated houses that are built with innovative prefabrication technologies and that are facilitated by BIM have been shown to reduce the carbon footprint of concrete and alleviate the environmental pressure caused by construction [55]. In addition, BIM-assisted analyses of building materials, such as concrete, steel, and cement, provide evidence to inform the development of policies and regulations to reduce the energy consumption and emissions [56,57].

In Cluster 4 (green color), the energy efficiency has a close relation to keywords such as “performance”, “design”, “modelling”, and “simulation”. The energy performance and efficiency simulation for a building using data from BIM can help to derive sustainable and sound decisions at the design stage, resulting in reduced energy consumption and carbon emissions [9,58,59,60,61,62]. The collaboration between BIM and other energy-design software can also yield key information for improving the energy efficiency [63]. In addition, BIM-based technologies can be used to evaluate the existing building solutions or retrofit solutions, thereby facilitating the development of net-zero-energy buildings (NZEBs) [64,65,66,67]. In addition to the design, the reliance on BIM technology aids in addressing the building energy efficiency in the operational stage, which contributes to the carbon-emission targets [68,69]. Although BIM offers new opportunities to improve the building energy efficiency and minimize carbon emissions, it has been argued that there is a need to upskill stakeholders, such as construction workers, through proper BIM education to meet the demands of the digital transformation of the construction industry [70].

The keywords (e.g., “energy”, “green buildings”, “policy”, “emissions”, “challenges”, and “governance”) are strongly connected in Cluster 5 (purple color). Although green buildings are more expensive to build upfront, they improve the energy consumption with the associated water efficiency, which saves operating costs and reduces the carbon footprint [71]. Green building has been considered one of the least costly approaches to mitigate climate change [72]. Different countries around the world have developed a variety of policies and measures to address the challenges of green-building development [45,73,74,75,76]. Although the incentives of policies can promote the use of green-building technology (GBT) in the building industry, the effect of multiple policies on reducing the carbon emissions from urban buildings is not the same as the associated effect of individual policies [72,77]. Assessing the long-term environmental benefits of multiple policies is essential for policy improvement and prioritization [78]. In terms of adjustment and climate change, the market-based policy mechanisms, such as carbon taxes and carbon trading, assist to achieve the carbon-emission targets while stabilizing industrial production [73].

Table 1 shows the top-10 keywords with co-occurrence strengths in the published articles regarding BIM and carbon emissions for sustainable buildings from 2008 to 2021 (14 years), which are: “BIM”; “life cycle assessment”; “green buildings”; “design”; “construction”; “carbon emissions”; “performance”; “sustainability”; “energy”; “residential buildings”. Keywords with higher connection strengths and frequencies have more influence. Design and construction are the stages of the building lifecycle, indicating that the current focus of BIM in the field of carbon emissions for sustainable buildings is on the early stages of the lifecycle: design and construction.

**Figure 5:**
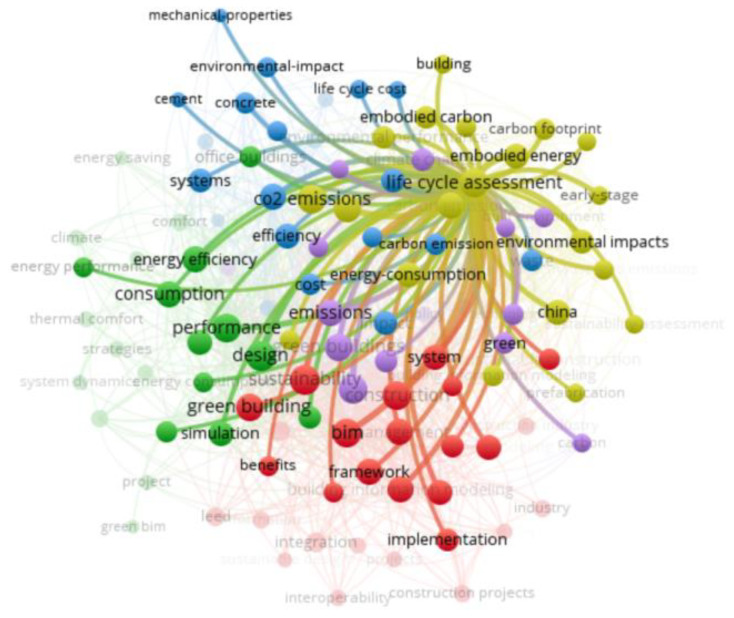
Network-visualization map with the theme of lifecycle assessment (further revealed in detail in Figure 4) in the WoS-core-collection database via VOSviewer software (generated by the authors).

“Life cycle assessment” is a significant node in Figure 4, which is further revealed in detail in Figure 5, with strong links to “BIM”, “design”, “construction”, “green building”, “energy”, “carbon emissions”, and “performance”, which suggests that the LCA is already well integrated in the field of sustainable design and construction. Design and construction are the focuses of the studies on the lifecycle, and they play significant roles in influencing the carbon emissions in sustainable buildings.

**Figure 6:**
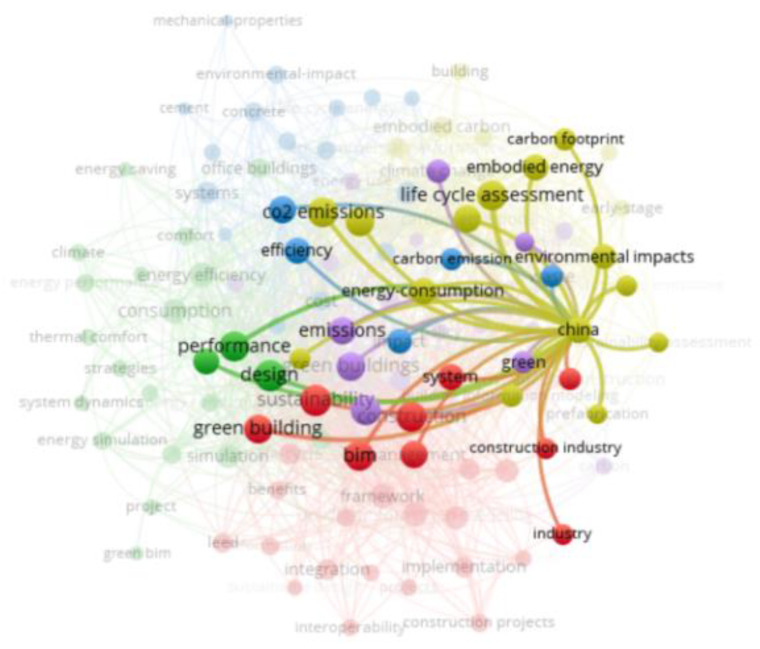
Network-visualization map with the theme of China (further highlighted in detail in Figure 4) in the WoS-core-collection database via VOSviewer software (generated by the authors).

Interestingly, “China” is the only keyword representing a country that appears as a significant node in the network-visualization map (Figure 4), which is further highlighted in detail in Figure 6. The keywords closely associated with China are “life cycle assessment”, “carbon emissions”, “green building”, “BIM”, “design”, and “construction”, which indicates that China has a certain influence in the field of BIM, carbon emissions, and sustainable buildings. China has been the world’s largest carbon emitter since 2006 [79], and it has now announced that it expects to reduce its carbon-emission level per unit of gross domestic product (GDP) to 60–65% of the year 2005 by the year 2030, and to achieve carbon peaking around 2030 [80]. The construction industry in China generates approximately 30% of the total carbon emissions, and it is therefore a key industry for achieving the national strategy targets on carbon emissions [81]. In addition, China has been actively promoting economic instruments, such as carbon emissions and carbon taxes, to promote the sustainable development of the construction industry [73,82]. In order to promote the adoption of GBT in construction, the Chinese government has introduced a series of industry standards and environmental-protection acts that support the development of green buildings and BIM [81,83,84]. In this context, green and sustainable buildings and BIM have significant market potential in China [17,85].

#### 3.2.2. CiteSpace Keyword-Burst Detection

**Figure 7:**
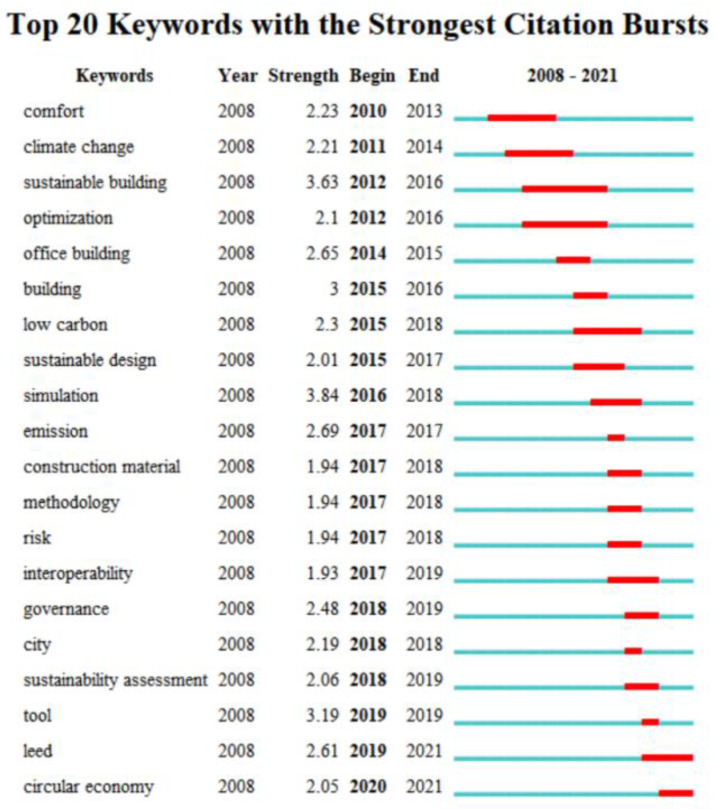
The 20 keywords with the strongest citation bursts in articles on BIM in the field of carbon emissions for sustainable buildings from 2008 to 2021 via CiteSpace software (generated by the authors).

Burst-detection analysis identifies and explores the frontiers of the research and latest trends in a particular field. Burst detection in CiteSpace is mainly based on Kleinberg’s algorithm [86], which identifies time periods in which a target trend is uncharacteristically frequent. A burst keyword is a keyword that suddenly increases in the number of references or occurrences within a certain period. Its basic principle is to identify hot words based on the growth rates of the frequencies of their occurrences. The time-dependent nature of these keywords is often considered to be at the forefront of the research in a particular field [87]. The detection of bursting keywords allows for obtaining research hotspots and predicting future research trends. The top-20 bursting keywords were obtained through CiteSpace. Figure 7 shows the top-20 keywords with the strongest citation bursts, of which the “keywords” represent the burst terms; “Year” represents the start of the analysis, which spans 2008–2021; “Strength” represents the intensity of the burst; “Begin” represents the year of the start of the keyword burst; “End” represents the year of the end of the burst; the red line represents the duration of the burst [88].

As shown in Figure 7, “sustainable building” and “optimization” became research hotspots in 2012, and this lasted for four years, which is the longest duration of all the bursting words. Sustainable buildings have huge potential to reduce the greenhouse effect [89]. Rising energy costs and the need for greater energy efficiency have raised the public awareness of the need to reduce the energy consumption throughout the lifecycles of buildings, and they have led to efforts to integrate green- and sustainable-building initiatives into the traditional building design, construction, and operation processes to optimize buildings towards sustainability [39]. “Simulation” was a research hotspot in 2016, which is the strongest (strength: 3.84) of the bursting keywords. Advances in information technology have enabled BIM to become an energy-simulation tool for early integrated building design [90]. In addition, the bursting keywords in 2021 are “LEED” and “circular economy”, which are also current research hotspots in the sustainable-building sector. The growing demand for sustainable buildings over the past few years has resulted in several countries establishing their own green-building rating systems. LEED is the most widely used building-assessment system worldwide, and it is used in several countries/regions, including the United States, Canada, Brazil, Mexico, India, and China [38,91]. The current research shows that the integration of BIM with LEED can speed up the LEED-certification process by assessing the sustainability of buildings at the design stage [37]. Due to the increasing demand for buildings to be environmentally friendly, some public-sector buildings are being required to use BIM from the design stage, which has led to an increasing number of building projects requiring both BIM and LEED [92]. The circular economy (CE) has great potential in the construction industry, which currently has an unmatched impact on the environment with the requirements of sustainable development [93]. The bursting keywords emerged in recent years for a relatively short periods of time, which indicates that the hotspots of research on BIM in field of carbon emissions for sustainable buildings have changed rapidly over time [94]. Studies have focused on climate change since 2011, after which sustainable buildings gained attention. In recent years, “government”, “cities”, “tools”, “LEED”, and “circular economy” have become hotspots, indicating that the carbon emissions in sustainable buildings have gradually improved, for which government policy plays an important role.

### 3.3. Microqualitative Analysis

In order to analyze the specific role of BIM in sustainable buildings in relation to carbon emissions, 81 articles closely related to BIM, sustainable buildings, and carbon emissions were selected for a microqualitative analysis from the 524 publications in the macroanalysis, and the articles were mapped across the building lifecycle stages, including the design, construction, operation, demolition, and full lifecycle. The selection of strong relevant literature was based on the following criteria: first, the articles dealt with the three knowledge concepts of BIM, sustainable buildings, and carbon emissions; second, the articles could be mapped to the stages of the building lifecycle for analysis.

As shown in Table 2, the mixed-research method [95,96,97,98,99] is mainly used in the design stage, followed by case analysis [9,60,100,101] and modeling [35,102,103], to explore the BIM decision-making model for reducing carbon emissions in sustainable buildings. The environmental impact associated with the design stage is up to 70% of the whole impact throughout the building lifecycle phases [104]. The integration of BIM with decision-making tools helps to address the difficulties of making sustainable-material decisions early in the design process [105]. BIM-assisted multiple-criteria decision making (MCDM) allows for an analysis of the key factors that affect the carbon emissions and energy efficiency in sustainable buildings [96], in which the MCDM allows alternatives to be evaluated and optimal decisions to be made [97,106]. By implementing BIM and LCA with a database, the environmental impacts of the design solutions can be measured at an early stage [103], which allows for a faster and more accurate quantification and assessment of the environmental impacts of different building materials for selecting the most sustainable building materials at the design stage [69,107]. A BIM-based approach to building design optimization can help with the tradeoff between the lifecycle cost (LCC) and lifecycle carbon emissions (LCCEs) of a building design, which aids designers to provide cost-effective and environmentally friendly design solutions [108]. In addition, the BIM-assisted LEED-certification system provides a framework to calculate the points that are earned at the concept stage for automatically assessing the sustainability of the building [37]. The integration of BIM and the LEED-certification process at the conceptual design stage also allows for the automatic calculation of the LEED-certification points to be compiled and the associated registration costs for the green and certified materials. In terms of energy use, the integration of BIM with energy-modeling packages enriches the energy analysis of the building, which leads to significant energy cost savings and reductions in electricity and carbon [9,61,63,95,101]. In addition, BIM enhances the quantitative assessment of the implied carbon emissions, and it optimizes the design at the building-element (BE) level, enabling low-carbon design concepts to really take hold [99]. The use of BIM offers various opportunities for integration with systems of building analysis and decision making [109], which provides building designers faster and more accurate approaches for design decision making that have a positive impact on the building carbon emissions and sustainability assessment of the building [64].

As shown in Table 3, the main research methods used in the construction phase of BIM-aided building carbon emissions are modeling [64,113,114,115] and case studies [116,117,118]. The refurbishment of buildings obtains attention at this stage. During the construction or renovation of a building, a large amount of energy and materials are used, and a large amount of carbon is produced [119]. Based on BIM, the carbon emissions during construction can be effectively assessed, which provides a good reference for the selection of low-carbon-emission materials. In addition, the longer the distance of the delivery of the construction material to the construction site, the greater the carbon emissions [114]. The integrated framework based on BIM and the web-map service (BIM–WMS) facilitates the selection of the construction-material suppliers and the planning of the material-transport routes [115]. Interestingly, refurbishment is effective at achieving a better energy performance to reduce carbon emissions [117]. A scan-to-BIM approach was used to assess the feasibility of retrofitting options for existing buildings [64]. Moreover, based on the BIM integration with energy-modeling software, the building energy performance can be optimized to determine the most energy-efficient and cost-effective strategy for the building renovation [117,118,120,121]. However, challenges remain in BIM for building renovations and retrofitting, with the data capture being the first and most critical issue for renovation projects [122]. With the vast number of metadata available from BIM models, the analysis of such building data using artificial-intelligence (AI) techniques offers new options for decision making based on continuous system learning [113]. This is important for deep refurbishment projects to improve the energy efficiency of buildings towards NZEBs.

As shown in Table 4, the research themes during the operations phase focus on the performance of the building operations and the tradeoffs between the implicit and operational energy, with modeling [44,68,116,126] and case studies [127,128,129] as the main research methods. The operation and maintenance stages take the longest and cost the most to the project owner compared with the other building lifecycle phases [130]. Studies have shown that the operating energy (OE) accounts for the major share of a building’s lifecycle energy use, followed by the embodied energy (EE), while other stages of the lifecycle consume less energy [131]. The increase in the global energy use demonstrates the urgent need to effectively and comprehensively reduce the energy and carbon footprints of buildings [132]. Reducing the OE could increase the EE, demonstrating the importance of the tradeoff between the OE and EE [133]. A BIM-driven design process can efficiently address the tradeoffs between specific and operational energy sources [127,134]. In addition, the use of BIM to assist the energy efficiency during the operation and maintenance phases of a project facilitates bridging the gap between the predicted and actual energy consumption of a building, which contributes to the goal of reducing the carbon footprint of the building [68].

As shown in Table 5, the impact of BIM on the carbon emissions during the building demolition phase has been less studied than in the other building lifecycle phases. The main themes of the studies focus on the greenhouse gases generated by construction waste, and the management and disposal of construction waste. The research methods for the studies in this phase are case studies [135], modeling [136], and reviews [50,137]. Based on BIM, it is possible to assess the carbon emissions of the building in the demolition phase during the building lifecycle, with the site-treatment phase being the largest contributor to the carbon emissions of the demolition phase [138]. Because the construction and demolition waste (CDW) end-of-life disposal process is a source of GHG emissions, the BIM-based quantification of CDW GHG emissions can lead to targeted GHG-reduction measures [136]. In addition, the application of BIM can increase the high recovery rate of CDW to achieve sustainable waste management [135]. Concrete has the highest emissions among the large amount of CDW, and asphalt has the highest CO_2_-emission capacity [136]. Furthermore, if concrete is recycled and reused at the end of its lifecycle, then the lifecycle of the GHG emissions can be reduced in the end-of-life phase [50].

As shown in Table 6, during the whole lifecycle, studies focus on the impact of BIM on the overall carbon emissions of sustainable buildings, and on the means of using the latest technology to assist in reducing the carbon emissions of buildings. This stage mainly adopts mixed-research methods [56,139,140,141,142,143,144], followed by modeling [40,51,145,146,147] and review methods [13,16,42,148,149]. BIM has been employed with green-building concepts (green BIM), which acts as a model-based process that generates and manages coherent building data throughout the project lifecycle to improve the building energy-efficiency performance and contribute to the achievement of the sustainable development goals [13]. Green BIM has a strong capability for holistic BIM-based green-building analysis, where the energy modeling and analysis can have a significant impact on determining the building performance in terms of the carbon emissions, energy use, sustainable-material selection, and cost savings [16]. BIM-based analyses for energy consumption that correspond to different orientations reveal that a well-oriented building can save significant amounts of energy throughout its lifecycle [150]. 5D BIM models allow for optimal decisions to be made regarding the appropriate energy and cost-effective envelope components [145]. Associating BIM modeling with LCA is the best procedure for achieving sustainable development and environmental protection, and for empowering the decision-making process in the building sector [15]. The approach can be used to determine which building elements are significant in the LCA. Furthermore, the integration of BIM and LCA allows for the assessment of the total carbon footprint of a building throughout its lifecycle, and it aids in completing the optimization of the greenhouse gas emissions throughout the lifecycle [4,18,51], for which the LCA is integrated with the BSA at an early stage of the project, based on the BIM approach. As such, designers can quickly assess the environmental impacts of their buildings while conducting concise sustainability assessments with few resources, addressing all the sustainability issues [141].

**Figure 8:**
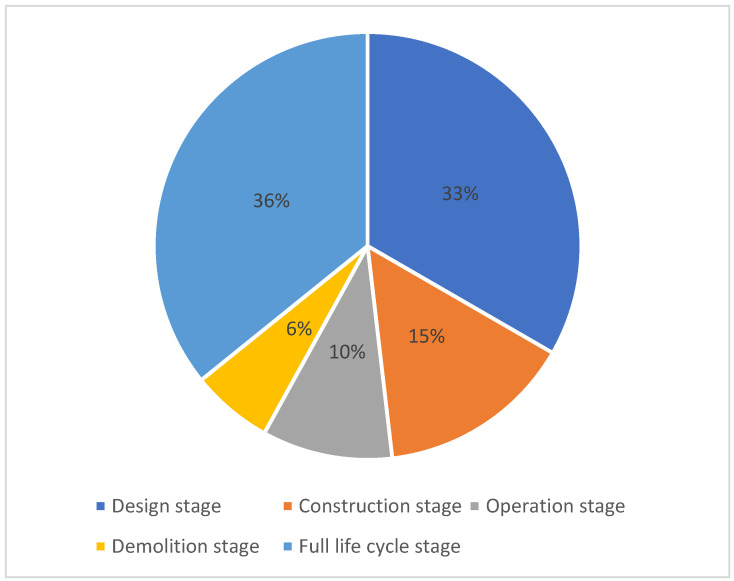
Weighting of publications in the field of BIM and carbon emissions for sustainable building during the building lifecycle for microqualitative analysis (generated by the authors).

As shown in Figure 8, research related to BIM around sustainable-building carbon emissions is concentrated in the full-building lifecycle stage and design stage, with the design and construction stages accounting for 48% of the overall microanalysis publications, and with only 16% of the publications focusing on the operational and demolition stages of the building. Although the nD capabilities of BIM make it potentially applicable throughout the full-building lifecycle phase, designers and contractors are primarily concerned with the application of BIM in the design- and construction-management stages. The capabilities of BIM are not well utilized in the operations, or in the demolition stages [149]. From an implementation-based value perspective, the implementing BIM shows a decreasing order of value throughout the lifecycle of a building [144]. Perhaps the wealth of the metadata that are available from BIM models opens up new possibilities for analyses at all stages of the building lifecycle [113]. The integration of BIM with a building management system (BMS) has the potential to improve the sustainability of the postconstruction phase of a building, which can go a long way to remedy the current deficiencies in the application of BIM [149]. In general, the integration of BIM and carbon-emission-enhanced frameworks will play a more important role in sustainability in the digitalization of the construction industry, in which the effective decision-making framework will help to achieve the goal of reducing carbon emissions [151].

## 4. Discussion

### 4.1. BIM Research Hotspots and Development Trends on Carbon Emissions within the Context of Sustainable Buildings

BIM is in a process of rapid development to facilitate strategies for carbon-emission reduction. This is mainly due to both policy and technical reasons. On the policy side, several countries/regions have taken effective measures to curb the carbon emissions that emanate from buildings [156], in which Chinese researchers have been very active and have achieved significant results, as indicated in the results of Section 3.2.1. It is interesting to note that, of the 524 publications included in the macro analysis, China contributed 168, or about 32%, of the total number of publications. To achieve environmental sustainability, China’s 12th and 13th Five-Year Plans have given significant impetus to the development of green buildings [157,158]. To achieve the goals of “carbon neutrality” and “carbon peaking”, China has introduced various legal measures and financial incentives in the 14th Five-Year Plan, which covers the years from 2021 to 2025, to improve the energy efficiency in high-carbon sectors using digital technology [159]. Studies have shown that BIM is becoming a key tool for the sustainable transformation of China’s construction industry [160,161]. China is therefore promoting the development of BIM in the field of sustainable buildings and carbon-emission reduction in many ways, including academic, policy, and technical.

The results of the burst-word detection suggest that LEED and the CE were the current hot policies that past studies were concerned with. There are currently a large number of LEED-registered and certified building projects internationally [162]. As green-building-assessment standards are crucial to the sustainable development of buildings, in recent years, there has been more and more attention paid to and studies on green-building-assessment tools [163]. There are studies that claim that LEED buildings contribute to reduced energy use, reduced carbon emissions, and greater human health benefits [164,165,166]. However, LEED requires appropriate monitoring and reporting mechanisms to ensure that it achieves its due design level. Otherwise, the actual performance of the building may not be as expected [167]. BIM-based capabilities can simplify the LEED-certification process and save time and resources [168]. The integration of BIM and value engineering can measure the relationship between the construction cost and energy saving while obtaining a LEED-compliant and cost-effective design solution [38]. Building owners and designers can benefit from the integration of BIM and LEED-certification systems to enable a sustainability assessment of the building process [37]. The CE and circular construction (CC) have received increasing attention over the past decade [169]. Research on the application of CE principles to the construction industry has grown in recent years [170]. The CE principles promote the maximum reuse of materials and minimize waste generation, leading to environmental and economic benefits [171,172]. Therefore, the CE assists in saving resources, and in reducing carbon footprints, the risk of the material supply, and price fluctuations [107,172]. Based on the accumulation of the building lifecycle information, BIM can provide effective decision making in the design phase of buildings based on the principles of the CE [173]. In the demolition stage of buildings, the use of BIM to reduce the construction waste has attracted more attention, which is instrumental for reducing carbon emissions and implementing the CE principles in buildings [174].

In terms of technology, the results of this research indicate that the development of LCA technology plays an important role in the impact of BIM on carbon emissions in sustainable buildings. BIM offers opportunities to improve the data transparency and compliance checks, as well as to automate LCA assessments [175]. The integration of BIM with LCA contributes to the advancement of the CE, as it enables the assessment of the entire lifecycle [107], which is a growing trend [176]. Although BIM and LCA integration can significantly reduce a building’s energy use and carbon footprint during the design phase, the potential of BIM and LCA for building carbon emissions in the other lifecycle phases has not yet been fully exploited. In the future, the integration of BIM and LCA is expected to play a more important role in carbon quantification and mitigation [43].

Furthermore, BIM is the first step towards the Industrial Revolution 4.0, of which digital twins and virtual reality are key elements [177]. BIM has been treated as a type of digital twin that offers the opportunity to integrate the physical and digital worlds, which greatly contributes to solving the industry’s challenges. As a result, over the past few years, researchers have been applying digital twins to solve industry problems, including those in the construction industry [178]. Although digital twins have being implemented in construction, most of the attention has been focused on the design and construction phases, neglecting the demolition and restoration phases [178]. In addition, BIM and various XR technologies (VR, AR, and MR) have shown great potential to change the way that the AEC industry designs, builds, operates, and monitors [179]. The metaverse based on the integration of XR and BIM enables the project remote collaboration of partners [180]. With the background of the coronavirus pandemic, the establishment of a building metaverse will pave the way for new opportunities in managing the carbon emissions of buildings. Furthermore, building intelligence is one of the future trends in the construction industry, which is a shift that requires the design, monitoring, and control of the data and information related to the energy assessment of the built environment through technologies such as BIM and digital twins. Thus, in the future, the digitization of the building sector will contribute significantly to the achievements of building energy, carbon-emission, and environmental-performance goals [141].

### 4.2. Challenges for the Use of BIM in Managing Carbon Emissions for Sustainable Buildings

The results of the microqualitative analysis indicate that BIM contributes to carbon-emission reduction throughout the lifecycle of a building, but the current studies mainly focus on the practice in the design phase, with less attention paid to the operation and demolition phases. This could be associated with the green-building-rating dilemma of carbon emissions, in which most certified green buildings are only effective in the design phase and perform unreliably in the operational phase [144].

There is a consensus in the literature that appropriate design decisions at the initial stage of a building project have a noticeable impact on the sustainability of a building, including the reduction in carbon emissions. Several studies report that holistic approaches through BIM facilitate the design of sustainable buildings, in which the integration of BIM with LCA or LEED promotes the selection of sustainable building materials and improves the building sustainability to reduce carbon emissions [37,69,107]. However, the interoperability is still the biggest challenge to BIM, and the integration of LCA with BIM suffers from a lack of data and difficulties in data exchange [37,47,107]. In addition, the most BIM-based LCA studies to date have focused on one-off assessments, rather than on iterative assessments in the building design process for sustainable decision making [181]. The cost of LEED in terms of BIM and LEED integration is expensive, and less and less owners are actively seeking LEED certification. The energy-performance tools that are part of the BIM paradigm can help with sustainable building decisions [37]. However, the tools used can influence the results and thereby produce poor decisions regarding carbon emissions [60].

During the construction phase, the BIM-based assessment of the carbon emissions provides a reliable reference for the selection of low-carbon-emitting materials [114]. Interestingly, refurbished buildings are more energy efficient, environmentally friendly, and cheaper than new buildings [182]. Although BIM has been widely used in new buildings, it is still in its infancy in building-refurbishment projects [119]. The assessment of the options for building refurbishment in terms of carbon emissions is needed to consider the energy-efficiency benefits of the building, the disruption caused to occupants, and the costs involved in the renovation process [117].

Studies have shown that the operations and maintenance phases have the most important role in the reduction in greenhouse gas emissions throughout the lifecycle of a building [44]. Currently, only a few studies have addressed BIM methods for improving the building energy efficiency and reducing carbon emissions during the operations phase. However, energy simulations performed in BIM software, such as Autodesk Revit, may not provide accurate results, as the simulation may not capture some of the data of the building, such as the heat-transfer pathways of the building [140].

In terms of the demolition phase, the studies are concerned with the disposal of construction-waste debris. Adopting sustainable deconstruction strategies, such as reuse and recycling, can also result in economic, energy, and carbon savings [148]. Additionally, the demolition phase is an increasing contributor to global greenhouse gases due to continued industrialization and urbanization [183]. However, the carbon emissions previously generated in construction and demolition waste have been largely ignored [138]. Furthermore, there are increasing concerns about the carbon emissions generated by the on-site collection and sorting during the recycling of demolition waste [138].

The assimilation of all the professional models and integrated facility data with BIM is very complex, and it is difficult to achieve data interoperability, as different BIM models from various disciplines are built via a variety of BIM software [143]. As such, the main challenges at present are the usability and model complexity of the BIM software specified for carbon emissions in green buildings, and the lack of interoperability across the BIM packages. Additionally, BIM faces the challenge of the penetration and learning costs [144]. From an economic perspective, BIM’s high initial investment costs and unpredictable returns have also hindered its development [184]. The lack of senior management attention also has a significant impact on the use of BIM in sustainable construction [185].

Building information models are considered to be the key components in future construction practice, in which their benefits for productivity and reliability are widely acknowledged. It is becoming increasingly important to use tangible performance data early in the design phase to influence the decisions and prevent errors on carbon emissions. Reusing existing BIM data repositories and operational building data can enable data-driven databases for sustainable building designs that aim to reduce carbon emissions [146].

## 5. Conclusions

This paper adopts a mixed-research method to quantitatively and qualitatively explore the current status, hotspots, challenges, and research trends in the application of BIM in the field of sustainable buildings that target carbon emissions. The research employed a bibliometric approach to obtain relevant studies by querying the WoS database with keywords, and it visualized the relationship between BIM, carbon emissions, and sustainable buildings through keyword co-occurrence via VOSviewer software, including network visualization and high-frequency keywording, and citation-burst detection via CiteSpace software for a macroanalysis from 2008 to 2021, followed by a microanalysis on the role of BIM in reducing carbon emissions during the sustainable-building lifecycle stages. The main contributions of this paper are as follows: (1) this is the first attempt to explore the relationship between BIM, carbon emissions, and sustainable buildings for the last 14 years (from 2008 to 2021) using bibliometric analysis; (2) this is the first use of a visualization software tool to analyze the trends, research hotspots, and applications of BIM to support carbon-emission reduction within the context of sustainable buildings; (3) compared with the existing studies, this paper presents a comprehensive analysis of the impact of BIM on reducing carbon emissions across the design, construction, operation, and demolition stages of buildings, in which the current research status in the field of each lifecycle stage was explored and critically analyzed. Sustainable construction, building lifecycle assessment, sustainable building materials, energy efficiency, and environmental strategies are the five most popular research directions of BIM that enables carbon-emission reduction. Moreover, this paper examined the policy and technology reasons for the rapid development of BIM for the reduction in carbon emissions for sustainable buildings. To meet the goals of the Paris Agreement, many countries have adopted policies and measures to achieve carbon neutrality in sustainable buildings by setting legislation, industry standards, carbon taxes, and carbon trading, for which BIM, as an important digital tool for the construction industry, plays an important role in the carbon emissions of sustainable buildings. However, from a technical point of view, there are still challenges, such as interoperability, in integrating BIM with LCA for an energy-efficiency simulation that results in carbon reduction. Furthermore, BIM, as a kind of digital twin in the construction industry, has a wide opportunity scope for development in carbon emissions. Sustainable buildings enhanced by emerging technologies, such as VR and AR, could be the key for the construction industry to open the gate towards a metaverse in the building and construction environment. Within the context of the pandemic, the current trend in metaverse development paves a way for BIM approaches in the field of carbon emissions for sustainable buildings. As such, the findings of this analytical research provide pointers to inform designers, builders, and policymakers in the development of BIM-driven carbon-emission-reduction strategies. This is timely considering the current political global carbon-neutrality target and the target of reducing carbon emissions by half by 2030, as well as the technical development of the emerging building metaverse. Although the current studies in the field have been developing rapidly, the studies mainly focus on the design stage. As such, the carbon-emission reduction through BIM in the later stages of the building lifecycle could be explored in future research. The bibliometric analysis in this paper was conducted based on the WoS-core-collection database. Future research could be extended to other databases, such as Scopus, to provide a collective view of the potential and value of BIM and emerging technologies, and especially the future building metaverse, in reducing carbon emissions in buildings.

## Figures and Tables

**Table 1:** High-frequency keywords of published articles regarding BIM in the field of carbon emissions for sustainable buildings from 2008 to 2021 (14 years) via the network visualization of VOSviewer software (generated by the authors).

Color ^1^	Cluster	Keyword	Occurrence	Total Link Strength
	1	BIM	173	723
	2	Lifecycle Assessment	103	541
	5	Green Buildings	127	475
	4	Design	86	455
	1	Construction	73	411
	2	Carbon Emissions	67	369
	4	Performance	76	362
	1	Sustainability	71	339
	5	Energy	61	295
	2	Residential Buildings	41	225

^1^ The colors in the table are in line with the colors in Figure 4.

**Table 2:** The role of BIM in the building design stage in terms of carbon emissions (generated by the authors).

Source	Year	Research Method	Research Topic
Barone, G [95]	2021	Model and case study	BIM and energy modeling
Haruna [96]	2021	Questionnaire and modeling	BIM and multicriteria-decision-making (MCDM) integration
Marzouk [97]	2021	Model and case study	BIM and MCDM integration
Khahro [9]	2021	Case study	Energy costs and carbon optimization
Xue [107]	2021	Literature review	Circular economy
Carvalho [98]	2021	Model and case study	BIM and building-sustainability-assessment (BSA) integration
Marrero, M [110]	2020	Modeling	Integration of BIM and lifecycle assessment (LCA)
Cang, YJ [99]	2020	Model and case study	Calculation of implied carbon emissions
Jalaei [37]	2020	Model and case study	BIM and Leadership in Energy and Environmental Design (LEED) integration
Wei [38]	2020	Model and case study	Building costs and energy efficiency
Galiano-Garrigos [60]	2019	Case study	Energy-performance and carbon-footprint assessment
Chen, SY [65]	2019	Model and case study	Net-zero-energy buildings (NZEBs)
Carvalho [100]	2019	Case study	BSA
Tushar [101]	2019	Case study	Energy-consumption optimization
Najjar [69]	2019	Model and case study	Integration of BIM and LCA
Singh [62]	2019	Case study	Building energy assessment
Eleftheriadis [111]	2018	Model and case study	Structural design optimization
Lee [61]	2018	Case study	Green BIM
Eleftheriadis [102]	2018	Modeling	Relationship between structural costs and carbon emissions
Akcay et al. [112]	2017	Model and case study	BIM and LEED integration
Chen et al. [106]	2016	Model and case study	BIM and MCDM integration
Liu et al. [108]	2015	Case study	The tradeoff between lifecycle cost (LCC) and lifecycle carbon emissions (LCCEs)
Jalaei, F et al. [36]	2015	Model and case study	BIM and LEED integration
Cemesova et al. [63]	2015	Case study	BIM and building-performance-simulation (BPS) integration
Jun et al. [35]	2015	Modeling	Green BIM template (GBT)
Jrade, A et al. [103]	2013	Modeling	Integration of BIM and LCA
Bank et al. [105]	2011	Modeling	BIM and system-dynamics (SD) integration

**Table 3:** The role of BIM in the construction stage in terms of carbon emissions (generated by the authors).

Source	Year	Research Method	New Build	Renovation	Research Topic
Zhao [64]	2021	Modeling		*	NZEBs
Mulero-Palencia [113]	2021	Modeling		*	Machine learning
Guo [123]	2021	Mixed		*	Green-building assessment and optimization
Piselli [120]	2020	Case study		*	Energy renovation of historic buildings
Sun [114]	2020	Modeling	*		Calculation of carbon emissions during construction
Chen [115]	2019	Modeling	*		Integration of BIM and web-map services (WMSs)
Edwards [119]	2019	Review		*	Sustainability decision making
Tzortzopoulos [117]	2019	Case study		*	Transformation program assessment
Ozarisoy [118]	2019	Model and case study		*	Low-energy design strategies
Hu [121]	2018	Model and case study		*	Educational building renovation
Kim [124]	2017	Modeling		*	Building energy optimization
Sattary [125]	2016	Model and case study	*		Bioclimatic principles

* indicates that the literature contains the content.

**Table 4:** The role of BIM in the operation and maintenance stages in terms of carbon emissions (generated by the authors).

Source	Year	Research Method	Research Topic
Venkatraj [134]	2020	Mixed	Tradeoffs between embodied and operational energy
Cheng [46]	2020	Case study	Integration of BIM and LCA
Piselli [116]	2020	Case study	Application of facility energy management
Chen [126]	2019	Case study	Workflow design
Shadram [127]	2018	Model and case study	Tradeoffs between embodied and operational energy
Petri [68]	2017	Case study	Building operations and energy performance
Costa [128]	2013	Modeling	Building operations and energy performance
Gokce [129]	2013	Model and case study	Energy-efficient building operations

**Table 5:** The role of BIM in the demolition stage in terms of carbon emissions (generated by the authors).

Source	Year	Research Method	Research Topic
Shi [135]	2021	Model and case study	Construction and demolition waste disposal technology
Li [137]	2020	Review	Construction and demolition waste management
Xu [136]	2019	Modeling	Greenhouse gas (GHG) emissions
Wang [138]	2018	Case study	Integration of BIM and LCA
Wu [50]	2014	Review	GHG emissions from concrete

**Table 6:** The role of BIM across whole lifecycle stages in terms of carbon emissions (generated by the authors).

Source	Year	Research Method	Research Topic
Gardezi [139]	2021	Model and case study	The relationship between physical characteristics and carbon footprint
Marzouk [151]	2021	Interviews	BIM and green-building assessment
Kurian [51]	2021	Modeling	Building carbon-footprint estimation
Li [56]	2021	Model and case study	Assembled concrete buildings
Figueiredo [140]	2021	Model and case study	Sustainable-material selection
Shukra [16]	2021	Review	Holistic green BIM
Carvalho [141]	2020	Model and case study	Integration of BIM and LCA
Fokaides [142]	2020	Mixed	Intelligent buildings
Dalla Mora [42]	2020	Review	Integration of BIM and LCA
Kaewunruen [143]	2020	Model and case study	Whole-life costs and carbon emissions
Wen [144]	2020	Mixed	BIM and green-building assessment
Montiel-Santiago [152]	2020	Model and case study	Sustainability and energy efficiency
Pucko [145]	2020	Modeling	Building envelope
Wang [4]	2020	Model and case study	Integration of BIM and LCA
Palumbo [48]	2020	Model and case study	Integration of BIM and LCA
Lu [18]	2019	Model and case study	Integration of BIM and LCA
Muller [148]	2019	Review	Interoperability of BIM
Petrova [146]	2019	Modeling	Data-driven sustainable design
Yang [45]	2018	Model and case study	Integration of BIM and LCA
Gan [153]	2018	Model and case study	A holistic BIM framework for low-carbon design
Marzouk [154]	2017	Model and case study	GHG calculations
Xie [40]	2017	Modeling	BIM and carbon calculations
Najjar [15]	2017	Model and case study	Integration of BIM and LCA
GhaffarianHoseini [149]	2017	Review	Postconstruction-energy-efficiency testing
Lu [57]	2017	Model and case study	Integration of BIM and LCA
Peng [155]	2016	Model and case study	BIM and carbon calculations
Abanda [150]	2016	Model and case study	The effect of the building orientation on the building energy consumption
Wong [13]	2015	Review	Green BIM
Lee [147]	2015	Modeling	BIM green template

## Data Availability

Publicly available datasets were analyzed in this study. These data can be found at https://login.webofknowledge.com/ (accessed on 2 January 2022).
